# Use of Terlipressin in AKI Associated with Hepatorenal Syndrome: CON

**DOI:** 10.34067/KID.0000000000000344

**Published:** 2024-06-27

**Authors:** Jamie Willows, Swapnil Hiremath

**Affiliations:** 1 Renal Services, Sunderland Royal Hospital, Sunderland, United Kingdom; 2 Department of Medicine, University of Ottawa and the Ottawa Hospital, Ottawa, Ontario, Canada

**Keywords:** AKI

Patients with hepatorenal syndrome (HRS) type 1 need better treatments—prognosis remains worse than most metastatic cancers. On this backdrop, the US Food and Drug Administration (FDA) have recently approved terlipressin. To briefly summarize, terlipressin is licensed for HRS with AKI in countries outside of North America (we will use the nomenclature of HRS-AKI, which is the new term for HRS type 1). However, the FDA were initially unconvinced by the data, meaning that the subsequent largest, highest-quality, randomized controlled trials (RCTs) were conducted by industry in the United States for licensing purposes—OT-0401 (*N*=112), Randomized, placebo-controlled, double-blind study to confirm the rEVERSal of hepatorenal syndromE type 1 with terlipressin (REVERSE, *N*=196) and most recently Study to Confirm Efficacy and Safety of Terlipressin in Hepatorenal Syndrome Type 1 (CONFIRM, *N*=300) *N* the largest ever HRS-AKI trial of any intervention).

We accept that the evidence is very strong that terlipressin plus intravenous albumin is much more efficacious in improving kidney function in HRS-AKI than midodrine and octreotide or using albumin alone. Terlipressin is not just a creatinine-lowering drug, and the evidence is also convincing that it improves the hard patient-centered outcomes of need for acute dialysis, as well as need for maintenance dialysis after liver transplant. There is potential for some patients to benefit from the FDA approval of terlipressin, but only if physicians are crystal clear on the potential harms.

## The CONFIRM Trial and Efficacy

The CONFIRM trial^1^ was an impressive effort in recruitment of critically unwell patients. The primary end point for reversal of HRS-AKI favored terlipressin at rate of 63/199 (32%) versus 17/101 (17%) with placebo. However, by day 30, ten patients with HRS reversal in the terlipressin group had experienced HRS recurrence^1^ (and five did not complete 30-day follow-up^2^) versus no HRS recurrences in the placebo group. Terlipressin was successful in increasing mean arterial pressure (MAP) by a mean of 3.4 mm Hg higher than the placebo group.^3^ However, starting MAP was relatively high at approximately 77 mm Hg. It might be that CONFIRM selected a population less likely to respond to vasoconstrictors because around 40% of patients had already been on midodrine–octreotide for ≥3 days. The subgroup analysis seems to show that those with baseline MAP <70 mm Hg may have more to gain from terlipressin.^1,3^

## The CONFIRM Trial and Respiratory Failure

The efficacy outcomes were overshadowed by fatal respiratory adverse events within 30 days, of 8.5% in the terlipressin arm versus 1% with placebo.^4^ Given CONFIRM randomized more patients to terlipressin than the previous two RCTs combined, clearer identification of safety signals is expected. It has been suggested in editorials^5^ that excessive albumin led to obscuring of benefit from terlipressin—but this is far from clear cut. *Post hoc* analysis^4^ demonstrate that the amount of albumin administered before randomization did not associate with risk of respiratory failure in CONFIRM. The gross excess of respiratory failure in the terlipressin group was despite more diuretic use in the terlipressin group at 25.5% versus 13.1%,^2^ the placebo group receiving larger doses of albumin both before and after randomization, and 17% of the terlipressin group getting no additional albumin after randomization. Of course, it is entirely reasonable to predict that patients also receiving a vasoconstrictor would need significantly less albumin, and the fact that less was received does not mean the volumes were reduced sufficiently to accommodate vasoconstriction. It is also plausible that point-of-care ultrasound could assist in the domain of fluid assessment in HRS-AKI, but this is unproven, and widespread expertise does not currently exist. It is worth remembering that the investigators in CONFIRM took a population that was predicted by the model for end-stage liver disease score to have over 70% mortality at 90 days and managed to decrease mortality to around 50%—and while learning lessons about the importance of monitoring fluid state is critical, improving on the care they provided will be no mean feat.

## Mortality

**Figure 1:**
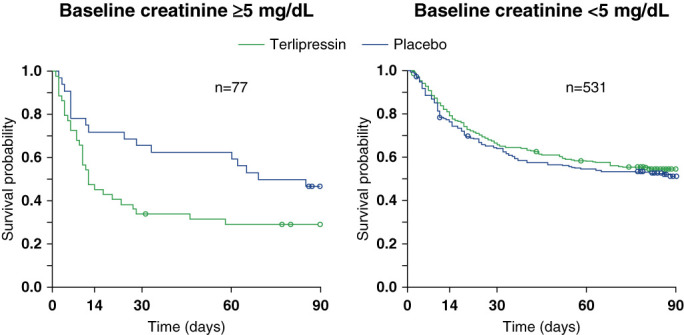
Overall survival from randomization to 90 days by treatment group and baseline serum creatinine, pooled analysis of OT-0401, REVERSE, and CONFIRM, modified for clarity only, from figure 30 in Mallinckrodt Pharmaceuticals Terlipressin Advisory Committee Briefing Document 5, which was provided without further statistical analysis.

CONFIRM was not powered to detect a mortality difference. Even if one summed OT-0401, REVERSE, and CONFIRM to total 598 participants, it would still be unreasonable to expect power for mortality (although the numbers are 48.7% mortality in the terlipressin group, 48.2% with placebo^3^). *Post hoc* analysis of the three large US terlipressin RCTs is concerning^3^. If starting serum creatinine is over 5 mg/dl, then patients seem to have survived better with placebo (Figure 1). The analysis of small subgroups can only be hypothesis-generating, but given patients in this group are also less likely to experience reversal of HRS-AKI,^3^ clinicians should exercise caution. It is possible that earlier treatment with effective vasoconstrictors would make them more effective, although future studies are needed to confirm this hypothesis. Some authors seem reassured that their European counterparts have used terlipressin for decades without clocking the excess mortality in the sickest patients, but in reality, this is just another call for humility. Very few patients with HRS-AKI do not receive terlipressin in Europe, and there is high mortality regardless of treatment, so no pattern would ever realistically be observed by even the most astute physician as is observed in large, pooled RCTs.

## Other Safety Signals

Despite the marketing pitch, terlipressin is not a precision medicine molecule, seeking out the splanchnic vasculature for isolated constriction. Instead, the off-target ischemic side effects of terlipressin are common, particularly affecting the bowel, coronaries, and peripheral circulation,^2^ and patients with coronary artery disease or peripheral vascular disease are particularly at risk. In addition, pooled analysis of OT-0401, REVERSE, and CONFIRM showed rates of sepsis or septic shock at 8.6% with terlipressin versus 2.4% with placebo.^2^ Although the mechanism of this is not immediately clear, this has led the European Medicines Agency to add a warning regarding increased sepsis with terlipressin use.^6^

Other new concerns also emerged. Although the terlipressin group in CONFIRM had more patients listed on the liver transplant list at baseline (28% versus 20%), they achieved a lower transplant rate than the placebo arm (23% versus 29%) at 90 days. Again, the trial was not powered to detect a difference, but given transplant is the definitive therapy option, this is clearly concerning. It is plausible that decrease in the mean model for end-stage liver disease by three points with terlipressin versus 1.1 points with placebo^3^ meant patients on terlipressin had lower priority on the transplant list on the basis of their current serum creatinine, which did not fully reflect their precarious clinical scenario.

## Alternatives

Currently, midodrine–octreotide is used on the floor of many US institutions for HRS-AKI. It has a much better safety profile than terlipressin, although this is likely because regarding efficacy, it has more in common with a homeopathic medicine than a genuine vasoconstrictor—real drugs have real side effects. By contrast, an effective vasoconstrictor-like noradrenaline is likely similar in efficacy to terlipressin. Current practice is for noradrenaline to be given in an intensive care setting. Trials with noradrenaline have not shown a signal for respiratory failure, although it is unknown whether this is due to an actual pharmacological difference, increased patient monitoring in ICU, or simply small sample size in the trials.

## Other Literature

Performing meta-analysis of terlipressin trials means including much lower-quality RCTs than the US studies mentioned above in the name of being comprehensive. For example, one such meta-analysis indicated mortality benefit from terlipressin.^7^ The three US RCTs were the only studies at low risk of bias and showed zero mortality benefit. For brevity, we will discuss only one example that was included: a single-center study^8^ that did not report inclusion or exclusion criteria, how randomization occurred, or any baseline vital signs or baseline biochemistry including serum creatinine or liver parameters and yet contributed greatly to the positive meta-analysis outcome. Similar concerns regarding quality arise in a trial which is the outlier in showing benefit of terlipressin over noradrenaline.^9^ This was single-center and open-label, compared not just agents but also strategies of vasoconstrictor uptitration with terlipressin titrated every 48 hours on the basis of serum creatinine and noradrenaline titrated every 4 hours on the basis of MAP and urine output (this difference is not discussed as a limitation), and had 0.23 mg/dl higher creatinine (unclear if mean or median) at randomization in the noradrenaline arm, that again they do not then discuss as a limitation. These confounded and biased observational real-world data,^10^ and editorials should not distract us from the robust evidence base from carefully conducted trials which also are conducted in the same real world.

## Terlipressin Mitigation Strategies

Much is made of the unblinded, 71-patient RCT which showed lower adverse events with an infusion instead of bolus regime of terlipressin.^11^ However, serious adverse events leading to drug withdrawal were still 21% in the infusion group (versus a huge 43% in the bolus group). Replication with less disconcerting withdrawal numbers would seem reasonable before infusion is touted as a savior mitigation strategy.

The FDA has approved terlipressin with a boxed warning^12^ about risk of serious or fatal respiratory failure and avoiding use when oxygen saturation <90%, incorporating the learning from CONFIRM. They rightly state that patients with acute-on-chronic liver failure grade 3 are at higher risk and within limitations of use that patients with serum creatinine >5 mg/dl are unlikely to experience benefit. Indeed, these patients would need to be exceptionally carefully chosen, ideally without cardiovascular comorbidities or recent hypoxia. Postmarket surveillance safety data must be collected and will need to be scrutinized carefully.

Terlipressin is not just coming to interested specialists; AKI in patients with cirrhosis is a common problem in every acute hospital, and frequently initial management of HRS-AKI falls to the generalist. In addition, withholding treatments from the sickest is a difficult challenge for humans to resist. There will inevitably be misuses as we try to build up experience with terlipressin, and the consequences of selecting the wrong patients are literally fatal.

Prognosis in HRS-AKI is dire even when underlying triggers are addressed, and terlipressin has some efficacy, so it is right that it is now available in the United States, although many unknowns persist regarding optimal patient selection. We hope that the postmarket surveillance safety report is robust, and a defense of humility remains vital for clinicians in the meantime. Importantly, it does seem that some subgroups do better with placebo than terlipressin, so efforts to find effective treatments in HRS-AKI must continue because no magic bullet is coming to US hospital floors which adequately fills the need. If unprecedented was the word of the pandemic, then judicious is the word of terlipressin editorials, and no doubt we will agree with the pro-terlipressin piece—judicious use is key.

## Supplementary Material
